# Thanking our peer reviewers

**DOI:** 10.1186/1476-4598-12-10

**Published:** 2013-03-01

**Authors:** Alan Storey

**Affiliations:** 1 Weatherall Institute of Molecular Medicine, University of Oxford, United Kingdom

## Abstract

**Contributing reviewers:**

As 2013 commences I would like to take a moment to reflect and recognize the peer reviewers that made the previous year possible. Listed below are those people who reviewed for *Molecular Cancer* last year. All are generous individuals who donated their time to assessing and improving our authors’ submissions. Your combined efforts have been invaluable to the editorial staff in maintaining the continued success of the journal in the Open Access forum.

The editors of *Molecular Cancer* would like to thank all the reviewers who contributed to the journal in Volume 11 (2012) by participating in the review process - taking time out of your busy schedules and even to volunteer - without your critical insights, hard work and support for the journal we wouldn’t be able to do what we do.

## 

Amir Abdollahi

Germany

Thomas E. Adrian

United Arab Emirates

Ricardo Aguiar

United States of America

Suresh Alahari

United States of America

Heike Allgayer

Germany

Suresh Ambudkar

United States of America

Nancy Amin

Australia

Peter Aplan

United States of America

William Arthur

United States of America

Hassan Ashktorab

United States of America

Wenlong Bai

United States of America

Nicholas Barker

Singapore

Betsy Barnes

United States of America

John Basile

United States of America

Alison Bauer

United States of America

Boon-Huat Bay

Singapore

Francesco Bertolini

Italy

Andrea Bertotti

Italy

Kapil Bhalla

United States of America

Feng Bi

China

Andrew Biankin

Australia

Eric Blair

United Kingdom

Massimo Bogliolo

Spain

Xandra Breakefield

United States of America

Massimo Broggini

Italy

Jacqueline Bromberg

United States of America

Robert Brown

United Kingdom

Matthias Buck

United States of America

Marie-Annick Buendia

France

Antony Burgess

Australia

Kerry Burnstein

United States of America

María Calzada García

Spain

Jennifer Carew

United States of America

Stacy Carl-McGrath

Germany

Amancio Carnero

Spain

Catherine Carpenter

United States of America

Jason Carroll

United Kingdom

Luis Carvajal Carmona

United Kingdom

Marc Chamberlain

United States of America

Paul Chapman

United States of America

Vasyl Chekhun

Ukraine

Srikumar Chellappan

United States of America

Wei-Jung Chen

Taiwan

Changyan Chen

United States of America

Robert Chen

United States of America

Benjamin Chen

United States of America

Yuhchyau Chen

United States of America

Nikki Cheng

United States of America

Liang Cheng

United States of America

Chun Cheng

China

Annie N.Y. Cheung

China

Francesca D. Ciccarelli

Italy

Clemente Cillo

Italy

Nicholas Clemons

Australia

Paolo Comoglio

Italy

Gerolama Condorelli

Italy

Simona Corso

Italy

Max Costa

United States of America

Doug Cress

United States of America

Richard Cummings

United States of America

Michal Dabrowski

Poland

Yan Dai

United States of America

Roger Daly

Australia

Pádraig D'Arcy

Sweden

Piyali Dasgupta

United States of America

Ruggero De Maria

Italy

Veerle De Vriendt

Belgium

Natasha Deane

United States of America

Thomas Decaens

France

Deepika Dhawan

United States of America

Maria Flavia Di Renzo

Italy

Francesco Di Virgilio

Italy

Song-Ze Ding

China

Yu Ding

United States of America

Bradley Doble

Canada

Shengli Dong

United States of America

Mark Drayson

United Kingdom

Stefan Duensing

Germany

Vincent Duronio

Canada

Wolfgang Eberhardt

Germany

Suzanne Eccles

United Kingdom

Mona Ahmed El-Bahrawy

United Kingdom

Douglas Faller

United States of America

Amir Fathi

United States of America

Francesco Feo

Italy

Cristiano Ferlini

United States of America

James Finke

United States of America

Warren Fiskus

United States of America

Matilde Y. Follo

Italy

Philippe Fort

France

Elizabeth Franzmann

United States of America

Simon Fricker

United States of America

Robin Fuchs-Young

United States of America

Simone Fulda

Germany

Imed Gallouzi

Canada

Paola Gasperini

Spain

Dirk Geerts

Netherlands

Robert Gemmill

United States of America

Nik Georgopoulos

United Kingdom

David Gewirtz

United States of America

John Glod

United States of America

Lee Goodglick

United States of America

Jeffrey Green

United States of America

Mark Greene

United States of America

Alex Gregorieff

Canada

Elizabeth Griffiths

United States of America

Donald Gullberg

Norway

Maolin Guo

United States of America

Sanjay Gupta

United States of America

Bernard Haendler

Germany

Gunnel Hallden

United Kingdom

Haiyong Han

United States of America

Zhaozhong Han

United States of America

Katherine Hannan

Austria

Yasuaki Harabuchi

Japan

T. Kendall Harden

United States of America

Malini Harigopal

United States of America

Elizabeth Harker

United States of America

Ping-an He

China

Carola Hedberg

Sweden

Danielle Heideman

Netherlands

Jozien Helleman

Netherlands

R. Scott Heller

Denmark

An Hendrix

Belgium

Andreas Herbst

Germany

Meenhard Herlyn

United States of America

Saw-See Hong

France

Miles Houslay

United Kingdom

Ji Fan Hu

United States of America

Qihong Huang

United States of America

Amandine Hurbin

France

Mohamad Hussein

United States of America

Yu Imamura

United States of America

M. Anwar Iqbal

United States of America

Claudio Isella

Italy

Sumiya Ishigami

Japan

Toshiyuki Ishiwata

Japan

Tae Jung Jang

Korea, South

Qing Jing

China

Frank Jones

United States of America

V. Craig Jordan

United States of America

Byoung Heon Kang

Korea, South

Stefan Kasper

Germany

Masaru Katoh

Japan

Sukhwinder Kaur

United States of America

Yona Keisari

Israel

Khandan Keyomarsi

United States of America

Robert Kinders

United States of America

Raj Kishore

United States of America

Lidija Klampfer

United States of America

Steven Knapper

United Kingdom

Hasan Korkaya

United States of America

Nina Korzeniewski

Germany

Oliver Krämer

Germany

Geoffrey Krystal

United States of America

Surinder Kumar

United States of America

Gopal Kundu

India

Roland Kwok

United States of America

Liang-Chuan Lai

Taiwan

Francois Lamoureux

France

Ted Lampidis

United States of America

Wallace Langdon

Australia

Alex Lange

United States of America

Silke Lassmann

Germany

Patrizia Lavia

Italy

Brian Law

United States of America

Charles Lawrie

United Kingdom

Keith Leppard

United Kingdom

Yuanyuan Li

United States of America

Yang Li

United States of America

Ji-Liang Li

United Kingdom

Jane Lian

United States of America

Massimo Libra

Italy

Jonathan Licht

United States of America

Biaoyang Lin

United States of America

J.Y. Lin

China

Noralane Lindor

United States of America

Peter Linsley

United States of America

Gaolin Liu

China

Mingyao Liu

United States of America

Xiao-Li Liu

China

Lawrence Loeb

United States of America

Joseph Loftus

United States of America

Bal Lokeshwar

United States of America

Shan Lu

United States of America

Stephanie Ma

Hong Kong

David MacEwan

United Kingdom

Daruka Mahadevan

United States of America

Nupam Mahajan

United States of America

Sami Malek

United States of America

Marcos Malumbres

Spain

Theo Mantamadiotis

Australia

John Mariadason

Australia

Andrea Mastro

United States of America

Grant McArthur

Australia

Peter McHugh

United Kingdom

Iain McNeish

United Kingdom

Enzo Medico

Italy

Ralph Meyer

United States of America

Carine Michiels

Belgium

Thomas Mikeska

Australia

Ian Mills

United Kingdom

Anirban Mitra

United States of America

Yin-Yuan Mo

United States of America

Anne Monks

United States of America

Gareth Morgan

United Kingdom

Justin Mott

United States of America

Markus Muenz

Germany

Pamela Munster

United States of America

Michihiro Mutoh

Japan

Burt Nabors

United States of America

Fumihiko Nakamura

United States of America

Takuro Nakamura

Japan

Nora Navone

United States of America

Matthias Nees

Finland

Shin-ichi Nishina

Japan

Kazuto Nishio

Japan

Paola Nistico

Italy

Rosa Noguera

Spain

Anja Nohe

United States of America

Fumio Nomura

Japan

Douglas Noonan

Italy

Hirohide Ohnishi

Japan

Ken Olaussen

France

Patricia Opresko

United States of America

Jon Oyer

United States of America

Sven Påhlman

Sweden

Moorthy Palanimuthu Ponnusamy

United States of America

Chinmay Panda

India

Philippe Pasero

France

Helen Pearson

Australia

Guang Peng

United States of America

Shuping Peng

China

Jose Perea

Spain

Christos Perisanidis

Austria

Jeffrey Peters

United States of America

Steffen Petersen

Portugal

Yuri Peterson

United States of America

Toby Phesse

Australia

Maria Catherine Pietanza

United States of America

Albrecht Piiper

Germany

Smitha Pillai

United States of America

Micheline Piquette-Miller

Canada

Terrence Piva

Australia

Osvaldo Podhajcer

Argentina

Lun-Xiu Qin

China

Satyanarayana Rachagani

United States of America

Robert Ramsay

Australia

Basabi Rana

United States of America

Annapoorni Rangarajan

India

Ashutosh Rao

United States of America

Peter Revill

Australia

Anna Riegel

United States of America

Gail Risbridger

Australia

Sabrina Ronen

United States of America

Karen Rother

Germany

Erik Sahai

United Kingdom

Ryuichi Sakai

Japan

David Salomon

United States of America

Jeffery Sample

United States of America

David Santamaria

Spain

Sabine Santucci

France

Vittorio Sartorelli

United States of America

Jeffrey Schelling

United States of America

Jens Schittenhelm

Germany

Johannes Schmid

Austria

Thies Schroeder

United States of America

Hildegard M. Schuller

United States of America

Anna Ivana Scovassi

Italy

Peter Searle

United Kingdom

Gregg Semenza

United States of America

Daniel Sepkovic

United States of America

Annalucia Serafino

Italy

Guido Serini

Italy

Mukund Seshadri

United States of America

Yongfeng Shang

China

Manisha Sharma

Australia

Malathy Shekhar

United States of America

Takashi Shingu

United States of America

Yow-Ling Shiue

Taiwan

Noam Shomron

Israel

Alison Sinclair

United Kingdom

Sandeep Singh

India

Pankaj Singh

United States of America

Keiran Smalley

United States of America

Tim Smith

United Kingdom

Peter Snijders

Netherlands

Claudio Sorio

Italy

E. Richard Stanley

United States of America

Giorgio Stassi

Italy

Georgios Stathopoulos

Greece

Hidekazu Suzuki

Japan

Attila Szanto

Hungary

Adam Szpechcinski

Poland

James Talmadge

United States of America

Tuomas Tammela

United States of America

Takuji Tanaka

Japan

Hiroaki Tanaka

Japan

Bor Luen Tang

Singapore

Maria Angeles Tapia-Laliena

Germany

Sophie Tartare-Deckert

France

Kathryn Taylor

United Kingdom

Christina Thirlwell

United Kingdom

Geraldine Thomas

United Kingdom

Yanis Tolstov

Germany

Wei-Min Tong

China

Ivo Touw

Netherlands

Tatsuya Tsurumi

Japan

Axel Ullrich

Germany

Neli Ulrich

Germany

T Upile

United Kingdom

Andre van Wijnen

United States of America

Gabor Varbiro

United Kingdom

Lyuba Varticovski

United States of America

Loredana Vecchione

Belgium

Julien Vignard

France

Peter K Vogt

United States of America

Harald von Melchner

Germany

Mei Wang

China

Weidong Wang

United States of America

Xiao Qi Wang

China

Timothy Wang

United States of America

Yubao Wang

United States of America

Kishore Wary

United States of America

Georg Weber

United States of America

Louis Weiner

United States of America

Bernard Weissman

United States of America

Ulrich Friedrich Wellner

Germany

Alana Welm

United States of America

Meir Wetzler

United States of America

Thomas Wex

Germany

Edward Whang

United States of America

Adrian Whitehouse

United Kingdom

Theresa Whiteside

United States of America

Frank Winkler

Germany

Yuliang Wu

Canada

Wei Wu

China

Shunbin Xiong

United States of America

Liang Xu

United States of America

Sanjay Yadav

India

Munekazu Yamakuchi

United States of America

Jilong Yang

China

Liang You

United States of America

Kurt S. Zaenker

Germany

Fenghuang Zhan

United States of America

Tao Zhang

China

Yu Zhang

United States of America

Xu Dong Zhang

Australia

Ralf Zwacka

Ireland

Wilbert Zwart

Netherlands

